# Severe localised granulomatosis with polyangiitis (Wegener’s granulomatosis) manifesting with extensive cranial nerve palsies and cranial diabetes insipidus: a case report and literature review

**DOI:** 10.1186/s12883-018-1058-8

**Published:** 2018-05-01

**Authors:** James E. Peters, Vivek Gupta, Ibtisam T. Saeed, Curtis Offiah, Ali S. M. Jawad

**Affiliations:** 1 Cardiovascular Epidemiology Unit, Department of Public Health and Primary Care, Worts Causeway, University of Cambridge, Cambridge, CB1 8RN UK; 2 Department of Rheumatology, The Royal London and Mile End Hospitals, Barts Health NHS Trust, Bancroft Road, London, E1 4DG UK; 3 Department of Histopathology, Queen’s Hospital, Rom Valley Road, Romford, RM7 0AG UK; 4 Department of Radiology, The Royal London Hospital, Barts Health NHS Trust, Whitechapel Road, London, E1 1BB UK

**Keywords:** Granulomatosis with polyangiitis, Wegener’s granulomatosis, Vasculitis, Diabetes insipidus, Pituitary, Collet-Sicard syndrome, Cavernous sinus syndrome, ANCA, Rituximab, Cyclophosphamide

## Abstract

**Background:**

Granulomatosis with polyangiitis (GPA, formerly Wegener’s granulomatosis) is a multisystem vasculitis of small- to medium-sized blood vessels. Cranial involvement can result in cranial nerve palsies and, rarely, pituitary infiltration.

**Case presentation:**

We describe the case of a 32 year-old woman with limited but severe GPA manifesting as progressive cranial nerve palsies and pituitary dysfunction. Our patient initially presented with localised ENT involvement, but despite treatment with methotrexate, she deteriorated. Granulomatous inflammatory tissue around the skull base resulted in cavernous sinus syndrome, facial nerve palsy, palsies of cranial nerves IX-XII (Collet-Sicard syndrome), and the rare complication of cranial diabetes insipidus due to pituitary infiltration. The glossopharyngeal, vagus and accessory nerve palsies resulted in severe dysphagia and she required nasogastric tube feeding. Her neurological deficits substantially improved with treatment including high dose corticosteroid, cyclophosphamide and rituximab.

**Conclusions:**

This case emphasises that serious morbidity can arise from localised cranial Wegener’s granulomatosis in the absence of systemic disease. In such cases intensive induction immunosuppression is required. Analysis of previously reported cases of pituitary involvement in GPA reveals that this rare complication predominantly affects female patients.

## Background

Granulomatosis with polyangiitis (GPA, formerly Wegener’s granulomatosis) is an autoimmune vasculitis of small- to medium-sized blood vessels. The Chapel Hill 2012 Consensus Criteria define GPA as “necrotizing granulomatous inflammation usually involving the upper and lower respiratory tract and necrotizing vasculitis affecting predominantly small to medium vessels” [1]. In their seminal 1954 review, Godman and Churg recognized that Wegener’s granulomatosis, microscopic polyangiitis, and Churg-Strauss syndrome shared certain clinico-pathological features, and comprised a subset of vasculitides distinct from ‘periarteritis nodosa’ (now polyarteritis nodosa) [2]. The discovery that anti-neutrophil cytoplasmic antibodies (ANCA) are present in over 90% of patients with GPA and microscopic polyangiitis, and approximately 30% of those with Churg-Strauss syndrome has led to these three clinical syndromes being classified under the umbrella term ‘ANCA-associated vasculitis’. Patients with GPA typically show a cytoplasmic pattern of ANCA staining on indirect immunofluorescence (‘cANCA pattern’), with antibodies directed against proteinase-3 (PR3), a neutrophil protease.

The generalised form of GPA described by Wegener is characterized histopathologically by the triad of necrotizing vasculitis, pauci-immune focal necrotizing glomerulonephritis and necrotizing granulomatous inflammation of the respiratory tract. This systemic form of the disease can be life-threatening, particularly in the presence of pulmonary haemorrhage or rapidly progressive crescenteric glomerulonephritis. Prior to the introduction of cyclophosphamide [3], mortality was over 80% [4]. Modern treatment involves intensive immunosuppressive therapy with high dose corticosteroids and cyclophosphamide or rituximab to induce remission [5, 6]. Once remission is achieved, patients are switched to less toxic maintenance immunosuppression, such as azathioprine and low dose glucocorticosteroid.

A limited form of Wegener’s granulomatosis, with lesions localised to the lung, was later identified [7]. Patients with limited (now generally referred to as ‘localised’) disease are more likely to be young, female and ANCA negative than those with generalised GPA [8]. Localised GPA typically manifests with middle ear and respiratory tract features such as otitis media, sinusitis, nasal collapse and airway stenosis [8, 9].

Here we describe a patient with localised but severe granulomatosis with polyangiitis (Wegener’s granulomatosis) manifesting with progressive cranial nerve palsies and pituitary dysfunction. This case emphasises that localised cranial Wegener’s granulomatosis can result in serious morbidity in the absence of systemic disease. In such cases intensive induction immunosuppression is required.

## Case presentation

Our patient, a white British female, presented at the age of 32 years with epistaxis, nasal congestion, recurrent bilateral otitis media and progressive hearing loss to an Ear, Nose and Throat (ENT) clinic at her local hospital. A year prior to this she had been fitted with bilateral hearing aids for hearing loss of unknown cause. She subsequently developed a left lower motor neurone facial palsy. A computed tomography (CT) scan showed a left nasopharyngeal mass and inflammatory changes in the middle ear and mastoid air cells. Nasolaryngoscopy revealed inflammation and ulceration of the left nasal turbinate, septum and posterior pharyngeal wall. She had never used cocaine. She underwent an exploratory mastoidectomy with grommet insertion.

**Fig. 1:**
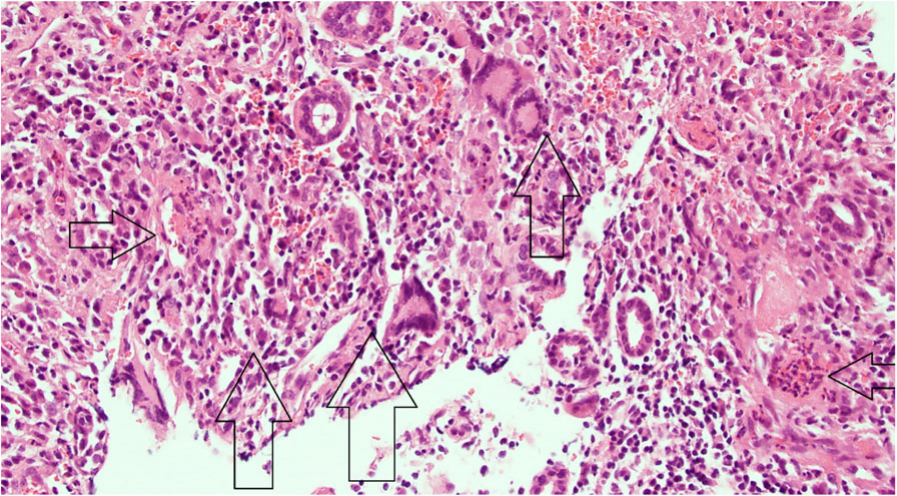
Histopathology. Nasal biopsy. H&E stain, magnification X100. Vertical arrows show granuloma. Horizontal left facing arrow: necrosis. Horizontal right facing arrow: angiitis with necrosis in vessel wall.

Biopsies of the right and left nasopharynx, left middle meatus and septum revealed florid acute and chronic inflammation with mucosal ulceration and extensive necrosis of stroma and cartilage. The inflammatory infiltrate consisted of neutrophils, lymphocytes, and plasma cells, with zones of histiocytes representing granulomata (Fig. 1). Large numbers of multinucleated giant cells were present. Eosinophils were inconspicuous. Histological assessment of vasculitis was difficult due to the extensive necrosis and marked tissue inflammation, but necrotizing vasculitis of small vessels was evident. There was no evidence of malignancy, and stains for fungi and mycobacteria were negative.

Anti-neutrophil cytoplasmic antibody (ANCA) testing was positive by immunofluorescence with a cytoplasmic staining pattern. Anti-proteinase-3 (PR3) antibodies measured by enzyme-linked immunosorbent assay (ELISA) were mildly elevated (5.8, normal range < 2). Anti-myeloperoxidase (MPO) antibodies were not detected. CT thorax revealed no pulmonary abnormality and no lymphadenopathy. Serum calcium and angiotensin-converting enzyme (ACE) were normal.

On the basis of the clinico-pathological findings, she was diagnosed with localised granulomatosis with polyangiitis (GPA, formerly known as Wegener’s granulomatosis). Detailed work-up revealed no evidence of pulmonary, renal or other systemic features. She was treated with methotrexate 25 mg weekly and prednisolone 60 mg daily. The prednisolone was weaned to 10 mg over a period of 6 months. As the steroid dose was reduced she deteriorated. She developed headaches, nausea, diplopia, dysphonia and progressive dysphagia to both solids and liquids, resulting in weight loss of 3 stone.

She was referred to our institution by her general practitioner approximately 1 year after her initial presentation. Physical examination revealed left ptosis, a dilated and unreactive left pupil, paresis of left eye movements consistent with palsies of cranial nerves III, IV and VI, a left lower motor neurone facial palsy, and deviation of the tongue to the right. There was wasting of the right sternocleidomastoid. Laryngoscopy revealed a right-sided vocal cord palsy. Blood tests showed mild elevation of the inflammatory markers; the erythrocyte sedimentation rate (ESR) was 34 mm/hour, and the C-reactive protein (CRP) was 26 mg/L. Renal function, urinalysis and urine microscopy were normal. Repeat ANCA testing revealed cytoplasmic pattern ANCA staining, but antibodies to PR3 and MPO were negative.

**Fig. 2:**
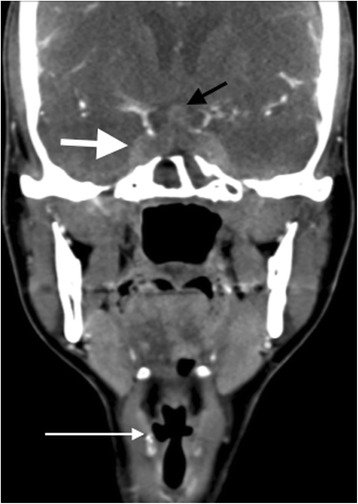
Coronal contrast-enhanced CT image of the skull base and neck (pre-cyclophosphamide treatment). There is marked abnormal chronic inflammatory infiltration of the cavernous sinuses bilaterally (bold white arrow) and hypophyseal involvement (black arrow). Multiple lower cranial nerve palsies (Collet-Sicard syndrome) as a result of the skull base inflammatory involvement were evident on imaging, including right vocal cord palsy indicated by adducted right true vocal cord and enlarged right laryngeal ventricle (long white arrow)

A contrast-enhanced CT scan of her head and neck (Fig. 2) demonstrated predominantly dural-based enhancing soft tissue abnormality affecting the central skull base. This chronic inflammatory tissue involved the sella turcica (with resultant erosion of the floor of the sella turcica) and both cavernous sinuses. There was associated abnormality involving the pituitary parenchyma, the pituitary stalk and the hypothalamus. Contiguous chronic inflammatory infiltrative changes were also evident in the orbital apices, the petrous temporal bones encasing the carotid canals and the extra-cranial infratemporal fossae including the pterygopalatine fossae. As a result of the cavernous sinus, petrous temporal bone and infratemporal fossae involvement, both internal carotid arteries were occluded in their skull base carotid canal segments, with the anterior circulation sustained through collateral vessels. The sinonasal region demonstrated chronic inflammatory features, with mucosal and submucosal fibrosis and ulceration. Bilateral chronic middle ear and mastoid inflammatory changes were evident. Radiological features of right Collet-Sicard syndrome (palsy of cranial nerves IX-XII) were also present. The appearances, particularly in relation to the skull base infiltration, had progressed significantly compared to 6 months earlier. Her pituitary hormone profile showed elevated prolactin (1353 munit/L; upper limit of normal range = 496) and reduced thyroid-stimulating hormone (TSH) (0.23 munit/L) with normal free thyroxine. On further enquiry, she reported amenorrhoea for the past year.

She had not yet had any children and was keen to do this in the future. We therefore initially opted for treatment with rituximab over cyclophosphamide. She received Rituximab 1 g, with a plan for a second dose two weeks later. Methotrexate was replaced with Azathioprine. She also received one pulse of intravenous (i.v.) methyl prednisolone 500 mg, and her oral prednisolone was increased from 10 mg to 30 mg. Given her modest baseline immunosuppressive treatment, and because of the insidious presentation and lack of systemic features, at the time we felt this mid-range prednisolone dose rather than 1 mg/kg was reasonable. Her CRP fell to 6 mg/L and she was discharged from hospital.

She was seen as an emergency 10 days later. She had developed complete dysphagia to solids and liquids and was unable to manage even small sips of water. Both pupils were now dilated and reacted sluggishly to light, and she had a new right-sided ptosis, indicating a new right-sided third nerve palsy. She also had a new right-sided sixth nerve palsy. Assessment of her swallowing showed that she was aspirating and had an ineffective cough reflex. She was kept nil-by-mouth, and fed through a nasogastric tube. The safety of her airway was assessed by the anaesthetic and ENT teams because of concern that she was at high risk of life-threatening airway obstruction or aspiration should the vocal cord palsies become bilateral. Her CRP had risen to 33 mg/L.

**Fig. 3:**
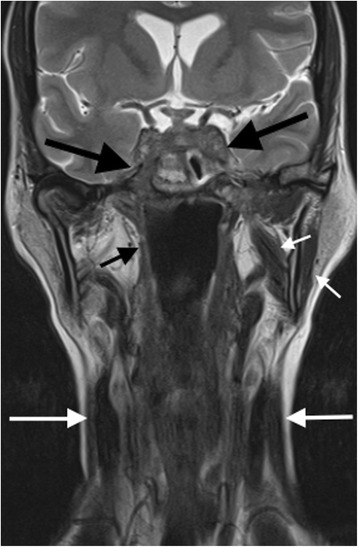
Coronal T2-weighted MRI image of the skull base and neck (pre-cyclophosphamide treatment). There is severe chronic inflammatory soft tissue abnormality affecting the cavernous sinuses and central and lateral skull base, including the left cavernous sinus and right sphenoid bone and foramen rotundum (large black arrows). Secondary cranial nerve palsies are present. Palsy of the mandibular division of the right trigeminal nerve has resulted in atrophy of the right masticator space muscle, in comparison to the more normal muscle volume on the left (short white arrows). Lower cranial nerve palsies are demonstrated by atrophy of the right superior pharyngeal constrictor muscle (short black arrow) and sternocleidomastoid muscles (long white arrows)

**Fig. 4:**
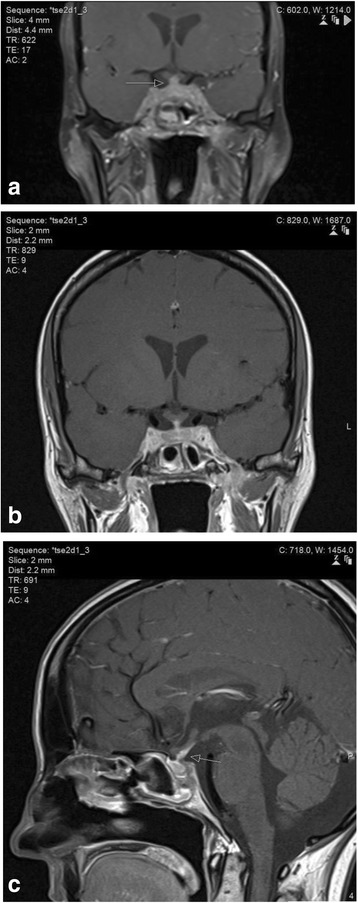
MR images of the brainstem, skull base and pituitary gland. (a) Coronal contrast-enhanced fat-saturated T1-weighted image of the central skull base demonstrates pathological enhancement of the cavernous sinus, pituitary gland and pituitary stalk (arrow). The abnormalities are non-discrete and radiologically inconsistent with a microadenoma or invasive macro adenoma of the pituitary gland. (b) Dedicated thin-section coronal contrast-enhanced T1-weighted image of the pituitary demonstrates the abnormal enhancement and thickening of the pituitary stalk as well as the cavernous sinus and pituitary parenchyma in more detail. (c) Dedicated thin-section sagittal contrast-enhanced T1-weighted image of the pituitary demonstrates the abnormal generalised thickening and enhancement of the pituitary stalk (arrow) consistent with granulomatous infundibulitis

A cranial magnetic resonance imaging (MRI) scan (Figs. 3 and 4a) confirmed the CT findings, and provided greater imaging sensitivity and specificity regarding the extent and severity of the osseus skull base infiltrative changes, the intracranial dural-based disease burden, and the cranial nerve palsies. Muscle atrophy due to denervation as a result of the latter was particularly well demonstrated. In addition, the MRI provided greater detail on the degree of hypothalamic-pituitary axis involvement, and allowed for subsequent imaging assessment of response to treatment.

**Fig. 5:**
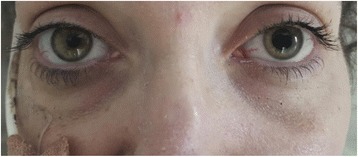
Photograph demonstrating dilated left pupil and nasal deformity. This was taken after 3 pulses of i.v. methyl prednisolone

She was treated with three pulses of i.v. methyl prednisolone 500 mg, with rapid improvement in her diplopia and normalization of the CRP. The right pupillary abnormality improved but the left pupil remained dilated (Fig. 5). Her oral prednisolone was increased to 60 mg, and she received three fortnightly doses of i.v. cyclophosphamide 750 mg (15 mg/kg) followed by three further doses at three-weekly intervals. Azathioprine was stopped. She also was given the second rituximab dose. She received topical mupirocin to reduce nasal *Staphylococcus* carriage and was started on oral co-trimoxazole both to reduce the risk of opportunistic infections and because this may reduce relapses in GPA [10]. Her swallowing remained compromised and she remained an inpatient for ongoing feeding via the nasogastric tube. Subsequently a percutaneous gastrostomy tube was inserted.

Her admission was complicated by hypernatraemia with serum sodium rising to 157 mmol/L. She required 4–5 L of fluid per day to maintain her sodium level in the normal range. She had two episodes of syncope due to postural hypotension. Her fluid balance chart revealed polyuria. Blood glucose and calcium were normal. Repeat dedicated pituitary MRI showed pathological enhancement and diffuse thickening of the pituitary (Fig. 4 b&c). She was diagnosed with cranial diabetes insipidus and treated with desmopressin, with normalization of her serum sodium. She was discharged home with feeding via the gastrostomy tube.

**Fig. 6:**
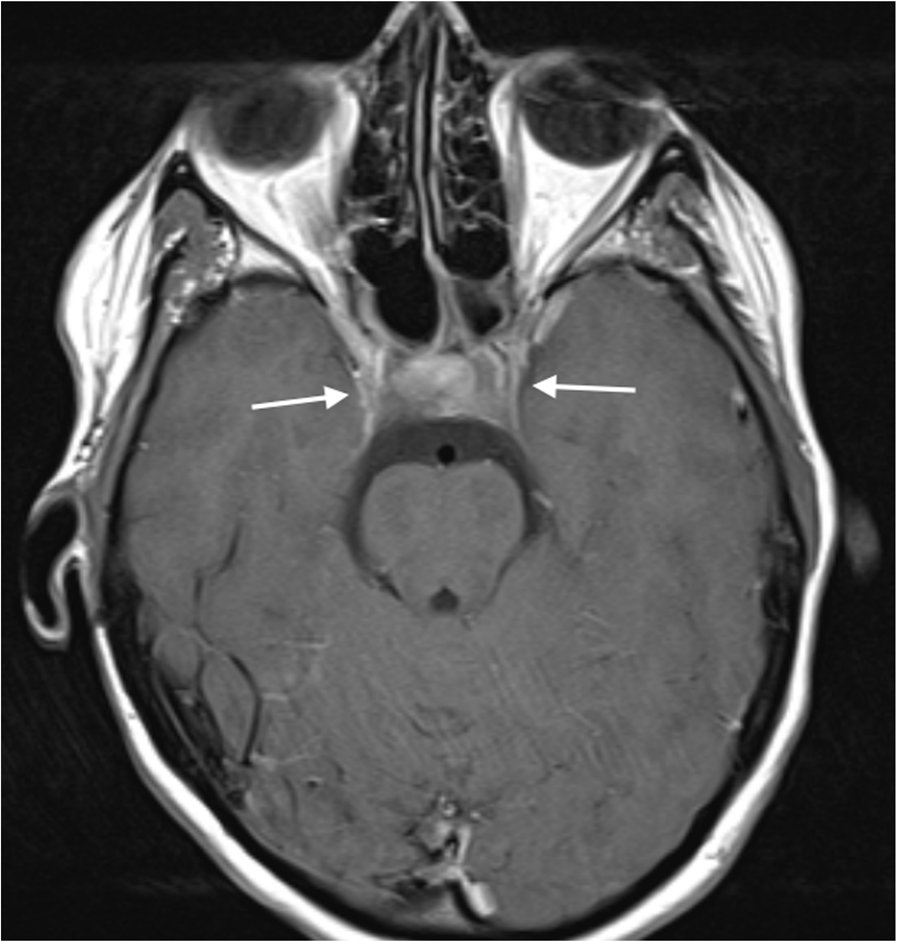
**Axial intracranial contrast-enhanced T1-weighted MR image after six pulses of cyclophosphamide.** There is reduction in the volume of abnormal cavernous sinus enhancing chronic inflammatory tissue (arrows) compared with pre-treatment imaging

After six pulses of cyclophosphamide, she showed marked clinical improvement. Her diplopia had resolved and eye movements had returned to normal. She was able to eat and drink normally. Her right-sided third nerve palsy had entirely resolved. Her left pupil remained dilated and reacted sluggishly to light, and a mild left facial weakness persisted. A further cranial MRI showed marked regression of the inflammatory changes in the skull base (Fig. 6). She was switched to maintenance therapy with azathioprine, and the prednisolone dose was slowly weaned to 5 mg daily. She remained clinically well on this treatment, with no progression of cranial disease on MRI, for 20 months following completion of cyclophosphamide. At this point, she had a relapse of ENT disease with nasal blockage and epistaxis requiring a tapering course of prednisolone 30 mg daily.

## Discussion

Our patient first presented with localised GPA confined to the nasopharynx and ear. Despite initial treatment with full-dose methotrexate and steroids, her disease progressed. She developed the unusual complications of extensive cranial nerve palsies (involvement of nerves II-IV, V_3_, and VI-XII) and diabetes insipidus due to granulomatous infiltration of the skull base.

Our report highlights several learning points. First, it demonstrates that localised GPA can be aggressive and result in serious morbidity. Assessment of disease severity is a key aspect of the management of AAV since it guides treatment decisions [5, 6]. Disease severity can be classified as life-threatening, organ-threatening (i.e. disease that will result in permanent tissue damage and/or loss of function unless appropriately treated), or non-organ threatening. Although our patient had anatomically localised cranial GPA, she developed organ-threatening disease with severe progressive cranial nerve involvement as a result of compression by inflammatory tissue. This included a vocal cord palsy, which resulted in dysphonia. If she had not been treated promptly, this could have become bilateral which can lead to life-threatening airway obstruction [11]. Glossopharyngeal, vagus and accessory nerve palsies resulted in severe dysphagia necessitating nasogastric feeding.

Second, this case illustrates central nervous system (CNS) manifestations of GPA, including the rare complication of cranial diabetes insipidus due to pituitary infiltration and compression. In contrast to peripheral nerve involvement, CNS disease in GPA is uncommon. In a European cohort of 128 GPA patients, 56 patients (44%) had peripheral neuropathy (either symmetrical polyneuropathy or mononeuritis multiplex), whilst only 9 patients (7%) had CNS involvement, of whom 6 had cranial nerve palsies [12]. CNS involvement occurred in 8% of cases in a cohort from the National Institutes of Health (NIH) [13], and cranial nerve palsies occurred in 21 of 324 patients (6%) attending the Mayo Clinic [14]. CNS disease in GPA is proposed to develop by three distinct but sometimes co-existing mechanisms [15]: by contiguous spread of granulomatous tissue from adjacent sites in the middle ear and sinuses (as in our patient), by primary granuloma formation within the CNS, or from vasculitis affecting the CNS. Seror et al. described three patterns of GPA-related CNS disease: chronic hypertrophic pachymeningitis, pituitary gland involvement, and cerebral vasculitis [16].

Yokoseki et al. reported a series of 36 patients with hypertrophic pachymengitis, of whom 17 had ANCA against MPO and 4 against PR3 [17]. Although the number of PR3 positive patients was small, this study found a higher frequency of pulmonary and renal disease in PR3 positive cases than in MPO+ cases, and that leptomeningeal and parenchymal involvement in the brain were more common in PR3 positive hypertrophic pachymeningitis compared with MPO positive and idiopathic hypertrophic pachymeningitis. In the presence of leptomengingeal AAV, cerebrospinal fluid analysis may reveal elevated protein and pleiocytosis, but these findings are non-specific. Other causes of hypertrophic pachymeningitis include other immune-mediated diseases (e.g. sarcoidosis and IgG4-related disease), infections, and neoplasms (particularly lymphoma) [17]. Lymphoma mimicking GPA with positive PR3 antibodies has been described [18]. However, in our patient, the radiological picture and the clinical course were not consistent with lymphoma.

Our patient had both chronic pachymeningitis and pituitary gland involvement, with meningeal thickening and pituitary infiltration evident on cranial imaging, and clinical and biochemical evidence of pituitary disease. Pituitary dysfunction due to GPA is rare, occurring in 9 of 819 patients (1.1%) in the French Vasculitis Study Group database [19], and 8 of 637 patients (1.3%) in a series from the Mayo Clinic [20]. We identified 58 previously reported cases of GPA with sellar involvement in the literature (Table 1), and analysed their clinical and demographic features. There was a significant female preponderance; 40 (69%) of the patients were female. Patients with pituitary involvement tended to be younger than GPA patients in general (median age at time of pituitary involvement 39.5 years, interquartile range 29–50 years). Pituitary involvement generally occurred early in the disease course. Of 46 cases where we were able to identify the age of onset of both GPA and pituitary involvement, in 28 (61%), pituitary disease was either the presenting feature or was diagnosed within a year of GPA onset. ENT involvement was the most common additional clinical GPA manifestation, occurring in 39 patients (67%). Other extra-CNS manifestations were, in order of decreasing frequency, pulmonary (*n* = 25), renal (*n* = 16), musculoskeletal (*n* = 15), ocular (*n* = 14), mucocutaneous (*n* = 12), peripheral neuropathy (*n* = 4), cardiac (*n* = 1), digital infarcts (*n* = 1), gastro-intestinal (*n* = 1). Diabetes insipidus was the most common endocrine abnormality, occurring in 47 patients (81%). In 4 of these, diabetes insipidus occurred following sellar surgery. Anterior pituitary hormone abnormalities occurred less frequently: hypogonadism was present in 32 patients, secondary hypothyroidism in 20, hyperprolactinaemia in 19, and growth hormone deficiency in 5. Reliable estimation of the frequency of adrenocorticotropic hormone (ACTH) deficiency was difficult due to therapeutic administration of exogenous corticosteroids in many cases. Typical MRI appearances include a sellar mass, sometimes with suprasellar extension, diffuse enlargement of the pituitary, thickening of the infundibulum (pituitary stalk), and enhancement with gadolinium contrast. Loss of the normal posterior pituitary hyperintensity on T1 weighted images was reported in 17 cases, and correlated with the presence of diabetes insipidus. In a few cases, hyperprolactinaemia and the presence of a pituitary lesion on imaging caused diagnostic confusion with a prolactinoma [21, 22]. However, diabetes insipidus and the loss of the normal high signal in the posterior pituitary on T1 weighted MRI are unusual in small pituitary adenomas. Our patient had diffuse pituitary and infundibular changes radiologically inconsistent with a pituitary adenoma.

**Table 1:** Previous reports of sellar involvement in GPA

1st Author & year	PMID	No. of cases	Age at GPA diagnosis	Age at time of sellar involvement	Sex	Pituitary Imaging (MRI unless stated)	Endocrinological abnormalities	Non-CNS features	ANCA
Haynes 1978 [29]	692550	1	26*	25	M	NA	DI Hyperprolactinaemia Normal TSH Normal LH & FSH	Lung, renal	N/A
Hurst 1983 [30]	6625709	1	47*	47	F	CT normal	DI Normal prolactin Normal TSH Post-menopausal LH/FSH	Polyarthritis, ENT, ocular, mucocutaneous, pulmonary	N/A
Lohr 1988 [31]	3172100	1	19*	19	F	Intrasellar mass	N/A	ENT, pulmonary	N/A
Rosete 1991 [32]	1865428	1	51*	51	F	CT: pituitary enlargement	DI	N/A	N/A
Czarnecki 1995 [33]	7611087	1	31	34	F	Sellar mass with suprasellar extension. Enhancement of the stalk and hypothalamus. Loss of PS.	DI Hyperprolactinaemia	ENT, arthralgia	N/A
Roberts 1995 [34]	7758239	2	71*	71	F	Intrasellar mass with suprasellar extension	DI (post-surgery) ↓ TSH ↓ LH & FSH ↓ cortisol Normal prolactin	None	cANCA +ve
			28*	28	F	Intrasellar mass with low-density centre	DI (post-surgery) Normal prolactin Normal TSH Normal LH & FSH Normal cortisol	Ocular, arthralgia, cutaneous, renal	cANCA +ve
Bertken 1997 [35]	9265867	1	36*	36	F	Macrocystic pituitary mass with suprasellar extension. Hydrocephalus	DI (post surgery) ↓ TSH response ↓ LH & FSH responses	ENT, pulmonary, ocular	ANCA -ve
Hajj-Ali 1999 [36]	10461488	1	N/A	21	F	Normal	DI	N/A	N/A
Katzman 1999 [21]	10219422	2	41*	41	F	Pituitary enlargement. Loss of PS	DI Hyperprolactinaemia Normal TSH Normal LH & FSH	Constitutional, arthralgia, ENT, ocular, cutaneous	cANCA +ve
			18*	18	F	Pituitary enlargement with contrast-enhancement. Loss of PS	DI Hyperprolactinaemia Normal TSH Normal LH & FSH	ENT, pulmonary	cANCA +ve
Miesen 1999 [37]	10069203	1	46*	45	M	Stalk thickening, contrast-enhancement. Loss of PS	DI Hyperprolactinaemia Hypogonadism Normal TSH Normal LH & FSH Normal cortisol & ACTH	ENT, renal, pulmonary	ANCA +ve
Goyal 2000 [38]	11003280	1	N/A (many years before pituitary involvement)	48	F	Sellar mass with suprasellar extension. Contrast-enhancement	DI ↓ TSH	Renal, pulmonary	cANCA +ve
Tappouni 2000 [39]	11096156	1	58*	57	F	Pituitary mass	DI	Constitutional, ENT, cutaneous, renal, pulmonary	PR3 +ve
Woywodt 2000 [40]	11028850	1	30*	30	M	N/A (diagnosed at autopsy)	N/A	N/A	N/A
Garovic 2001 [41]	11136194	1	47*	47	F	Cystic enlargement of the pituitary. Non-enhancing with gadolinium	DI ↓ prolactin ↓ TSH ↓ gonadotropins	Constitutional, cutaneous, pulmonary	cANCA +ve
Tao 2003 [42]	14642162	1	N/A	19	F	Pituitary and stalk enlargement with heterogenous enhancement	DI ↓ TSH Hypogonadism	N/A	N/A
Muir 2004 [43]	15150009	1	13*	13	M	Diffuse pituitary enlargement. Foci of ↑ T1 signal. More extensive ↑ T2 signal. Central contrast-enhancement. Loss of PS.	DI	ENT, pulmonary	ANCA +ve
Vittaz 2004 [44]	15687906	2	45	47	M	Pituitary mass, with contrast-enhancement. Pituitary stalk thickened & infiltrated. Loss of PS	Hyperprolactinaemia ↓ testosterone ↓ LH, normal FSH ↓ cortisol & ACTH Normal TSH	Constitutional, polyarthritis, peripheral neuropathy, pulmonary	PR3 +ve
			46	50	F	Pituitary enlargement	DI Hyperprolactinaemia Hypogonadism	Polyarthritis, ocular, mucocutaneous, ENT	ANCA +ve
Duzgun 2005 [45]	15864593	1	47*	47	F	Loss of PS	DI Anterior pituitary hormones normal	Polyarthritis, pulmonary, ENT, renal	PR3 +ve
Seror 2006 [16]	16523054	3	45	50	F	Nodular pituitary enlargement. Homogenous contrast-enhancement. Loss of PS	DI Hyperprolactinaemia Thyrotropic deficiency Hypogonadism Corticotropic deficiency	ENT, ocular, mucocutaneous	PR3 +ve
			26	41	F	Nodular pituitary enlargement. Contrast- enhancement	DI Normal anterior pituitary hormones	ENT, arthralgia, ocular, renal	PR3 +ve
			55	57	M	Pituitary enlargement & central necrosis. Heterogenous enhancement.	DI Hyperprolactinaemia Panhypopituitarism	Peripheral neuropathy, pulmonary, retinal vasculitis, digital, cerebral and renal infarcts	PR3 +ve
Spisek 2006 [46]	16322901	1	30*	29	M	Sellar cystic lesion	DI ↓ TSH ↓ LH & FSH ↓ ACTH ↓ IGF-1	ENT	PR3 +ve
McIntyre 2007 [47]	17318440	1	22*	22	F	Heterogeneous enhancing pituitary mass	DI Hypogonadism	Cutaneous, ocular, renal	PR3 +ve
Thiryayi 2007 [48]	17188492	1	21*	21	F	Sellar mass with central hypo-intensity	DI (post-surgery) Hypogonadism (post-surgery)	Constitutional, arthralgia	cANCA +ve
Yong 2008 [49]	17492510	1	33*	33	M	Pituitary stalk thickening. Contrast-enhancing nodule at the superior aspect of the stalk. Loss of PS.	DI Hypogonadism ↓ ACTH Normal prolactin Normal TSH Normal IGF-1	ENT	PR3 +ve
Cunnington 2009 [50]	20107566	3	19	24	M	Pituitary enlargement	DI Anterior pituitary hormones normal	Constitutional, ENT, pulmonary, ocular, cutaneous	PR3 +ve
			33	34	F	Diffusely enlarged gland containing a poorly enhancing lesion with supra-sellar extension. Loss of PS.	DI Normal prolactin Normal TSH	Constitutional, ENT	cANCA+ve
			26	35	M	Diffusely enlarged pituitary and thickened stalk.	DI ↓ TSH	Constitutional, ENT, pulmonary	cANCA +ve
Xue 2009 [51]	19172275	1	63*	63	F	Normal	DI TSH normal LH & FSH normal	Constitutional, pulmonary, peripheral neuropathy	PR3 +ve
Barlas 2011 [52]	21116602	1	35	37	F	Anterior enlargement. Central area with low signal on T1 and high signal on T2. Post contrast enhancement of pituitary and stalk. Loss of PS.	DI Hyperprolactinaemia	ENT, pulmonary, polyarthritis	cANCA +ve
Santoro 2011 [53]	22147097	1	53*	53	F	Hypointensity of adenohypophysis on T1. Hyperintense sectors on R side on T2. Peripheral contrast-enhancement. Stalk-thickening. Loss of PS.	DI ↓ TSH Hypogonadism Normal prolactin	Polyarthritis, cutaneous, pulmonary, renal	cANCA +ve
Tenorio- Jimenez 2011 [54]	22673710	1	23	38	F	MRI: marked infundibular thickening, sellar mass with hypointensity on T1. Loss of PS	Hyperprolactinaemia ↓ TSH Hypogonadism Post pituitary hormones unaffected	ENT, pulmonary, renal	cANCA +ve (−ve by the time of sellar manifestations)
Hughes 2013 [55]	23186961	1	N/A	30	F	Sellar mass	Panhypopituitarism	Ocular	N/A
Pereira 2013 [56]	22898089	1	48*	48	F	Appearances of pituitary microadenoma, but histopathlogy revealed necrotizing granulomatous inflammation	Hyperprolactinaemia ↓ TSH Post pituitary hormones unaffected	ENT	N/A
Kapoor 2014 [20]	25077899	8	N/A	67	F	Peripherally enhancing cystic sellar mass compressing the stalk	Hyperprolactinaemia Hypogonadism Normal TSH	ENT, renal	7/8 cases PR3 +ve
			N/A	48	F	Multiple non-enhancing cystic areas in the pituitary, convexity of superior margin of pituitary	DI ↓ prolactin ↓ TSH Hypogonadism Corticotropic deficiency	ENT, pulmonary, cutaneous	
			N/A	28	F	Sellar mass with large zone of central non-enhancement and peripheral enhancement. Stalk displacement	DI Hypogonadism Normal prolactin Normal TSH Normal IGF-1 Normal cortisol	ENT, pulmonary, renal	
			N/A	55	M	Sellar mass with suprasellar extension	DI Hypogonadism Normal prolactin Normal TSH Normal cortisol Normal IGF-1	ENT, pulmonary, renal, cutaneous, joints	
			N/A	35	M	Necrotic sellar mass with peripheral enhancement & suprasellar extension. Thickened contrast-enhancing stalk	DI ↓ TSH Hypogonadism Normal prolactin Normal cortisol Normal IGF-1	ENT	
			N/A	54	M	Enlarged pituitary measuring 12 mm, with heterogeneous enhancement. Slight diffuse thickening of the stalk	DI ↓ TSH Hypogonadism Normal prolactin Normal IGF-1	ENT, pulmonary, renal, cardiac	
			N/A	68	M	Homogeneously enhancing sellar mass, extending into the cavernous sinus bilaterally. Stalk preserved.	↓ prolactin ↓ TSH Hypogonadism ↓ IGF-1	ENT, joints	
			N/A	28	F	Sellar mass extending into the suprasellar cistern, with low T2 signal in the periphery and a bright centre. Peripheral enhancement with central cystic change. Thickening of pituitary stalk	DI Normal prolactin Normal TSH Normal cortisol Normal IGF-1	ENT	
De Parisot 2015 [19]	25906106	9	46*	46	F	Enlarged posterior pituitary. Infiltration of posterior pituitary. Loss of PS	DI	ENT, ocular	
			60	70	M	Normal	↓ TSH Hypogonadism ↓ IGF-1 Normal prolactin	ENT, peripheral neuropathy	
			23	24	F	Enlarged pituitary. Irregularity of infundibulum. Heterogeneous enhancement of anterior pituitary. Loss of PS	DI ↓ TSH Hypogonadism Normal prolactin Normal IGF-1	ENT	
			24*	24	M	Enlarged infundibulum	DI Hyperprolactinaemia ↓ TSH Hypogonadism Normal IGF-1	Renal, ocular, joints, gastro-intestinal	
			66	77	M	Enlarged pituitary. Stalk infiltration. Loss of PS.	↓ TSH Hypogonadism ↓ IGF-1 Normal prolactin	None	
			67	68	F	Normal	DI Hyperprolactinaemia Normal TSH Normal LH & FSH Normal IGF-1	ENT	
			28	42	F	Heterogeneous enhancement of pituitary	DI Hyperprolactinaemia Hypogonadism ↓ ACTH	ENT, lung	
			55	57	M	Sellar mass, heterogeneous. Enhancement. Enlargement and infiltration of stalk. Loss of PS	DI Hyperprolactinaemia ↓ TSH Hypogonadism ↓ ACTH	Pulmonary	
			46	50	F	Enlargement and infiltration of pituitary. Heterogeneous enhancement, contact with optic chiasm	DI Hyperprolactinaemia Hypogonadism Normal TSH Normal IGF-1	ENT, ocular	
Eli 2016 [22]	27521731	1	32*	29	F	Homogenously enhancing sellar mass. Thickened stalk.	Hyperprolactinaemia	ENT, pulmonary	MPO +ve
Esposito 2017 [57]	28540625	3	37*	37	F	Sellar mass extending into the suprasellar cistern with peripheral enhancement and central cystic lesion. Stalk deviation.	DI Hypogonadism GH deficiency	Constitutional, ENT	PR3 +ve
			36*	36	F	Cystic pituitary mass	DI Anterior pituitary function normal	Constitutional, myalgia, ENT, pulmonary	PR3 +ve
			32*	32	F	Sellar mass with homogeneous. Thickening of the pituitary stalk	DI Anterior pituitary function normal	ENT	PR3 +ve

Abbreviations: *ACTH* adrenocorticotropic hormone, *cANCA* cytoplasmic pattern ANCA staining, *DI* diabetes insipidus, *GH* growth hormone, *IGF-1* insulin-like growth factor 1, *LH* luteinising hormone, *FSH* follicular stimulating hormone, *MPO* myeloperoxidase, *N/A* data not available, *PMID* PubMed ID, *PR3* proteinase-3, *PS* posterior signal.

*indicates cases where pituitary involvement was diagnosed prior to or at the time of GPA diagnosis

For the purposes of the Table, a TSH within the reference range but inappropriately low for the T4 has been included in the category “↓ TSH”

The third issue raised by this case is the wisdom of methotrexate treatment at the time of her initial presentation. The NORAM trial compared methotrexate with cyclophosphamide for the treatment of early, non-organ threatening systemic disease [23]. Whilst there was no significant difference in the primary endpoint of overall remission rates at 6 months, relapse rate at 18 months was higher in the methotrexate group, and there was delayed remission in the methotrexate group in patients with more extensive disease or pulmonary involvement. The lack of a difference between methotrexate and cyclophosphamide at 6 months might reflect the fact that patients in both treatment groups were still receiving steroids at this timepoint. Post-trial follow-up has revealed that patients in the methotrexate arm of the trial received more corticosteroids, cyclophosphamide, and other immunosuppressive agents than those in the cyclophosphamide group [24], indicating less effective disease control with methotrexate compared to cyclophosphamide induction therapy. In light of these data and our clinical experience, it is the authors’ personal view that methotrexate should be used for only the mildest cases of GPA, and we have a low threshold for switching to stronger immunosuppressive therapy in the face of active disease. This view is supported by the 2015 EULAR guidelines which reserve methotrexate for the treatment of non-organ threatening disease [6]. The EULAR guidelines specifically spell out that methotrexate is not suitable therapy for meningeal disease, or for nasal/paranasal disease with bony involvement, cartilage collapse or deafness, and thus do not support the use of methotrexate in this patient, even at the time of her initial presentation. The absence of pulmonary or renal disease and the perception that localised disease is benign may have contributed to her initial under-treatment, and the failure to escalate immunosuppression at her local hospital.

The final issue is the choice of rituximab versus cyclophosphamide. We initially opted for rituximab over cyclophosphamide to reduce the risk of infertility. However, in view of rapid clinical deterioration we commenced cyclophosphamide. The RAVE and RITUXIVAS studies both showed non-inferiority of rituximab compared to cyclophosphamide in AAV [25, 26]. However, the results from these trials cannot necessarily be extrapolated to our patient. In the RITUXIVAS trial all patients had glomerulonephritis. The RAVE study was not restricted to patients with renal involvement, but did not have any patients with cranial nerve palsies in the rituximab treatment arm. Nevertheless, case series suggest that rituximab can be effective in cranial GPA [27, 28]. It should be highlighted that rituximab has a relatively slow onset of action, and in retrospect our initial “bridging” steroid therapy (one pulse of 500 mg iv methyl prednisolone and 30 mg oral prednisolone) was insufficient. The patient improved markedly after we commenced high-dose corticosteroids and cyclophosphamide. For severe cases where rapid control of disease is necessary, this combination of cyclophosphamide and rituximab may be preferable to either drug alone. Indeed, in the RITUXIVAS trial i.v. cyclophosphamide 15 mg/kg was given alongside the rituximab doses in weeks 1 and 3 [26].

## Conclusions

Pituitary involvement is a rare complication of GPA. It disproportionately affects female patients and tends to occur early in the disease course. Diabetes insipidus is the most commonly associated endocrinological abnormality. Our case report highlights that localised GPA is not necessarily indolent and that methotrexate may be inadequate treatment for localised disease. The presence of adverse features such as neurological involvement requires prompt recognition and aggressive therapy. Organ- or even life-threatening disease can occur in the absence of pulmonary or renal involvement. In this case a good outcome was achieved with high-dose steroid and dual treatment with cyclophosphamide and rituximab with minimal adverse events. In cases of severe neurological compromise due to GPA where a rapid therapeutic effect is required, such combination therapy may be a useful strategy. Finally, such cases should be managed by a multi-disciplinary team in a tertiary referral centre.

## Abbreviations

ACE: Angiotensin-converting enzyme
ACTH: Adrenocorticotropic hormone
CNS: central nervous system
CRP: C reactive protein
CT: Computed tomography
ELISA: Enzyme-linked immunosorbent assay
ENT: Ear, nose and throat
ESR: Erythrocyte sedimentation rate
EULAR: European League Against Rheumatism
GPA: Granulomatosis with polyangiitis
MPO: Myeloperoxidase
MRI: Magnetic resonance imaging
NIH: National Institutes of Health
PR3: Proteinase-3
TSH: Thyroid-stimulating hormone
